# Cervical paragangliomas: experience of 114 cases in 14 years

**DOI:** 10.1016/j.bjorl.2018.05.001

**Published:** 2018-06-11

**Authors:** Halil Basel, Nazim Bozan

**Affiliations:** a Lokman Hekim University Faculty of Medicine, Department of Cardiovascular Surgery, Ankara, Turkey; b Yuzuncu Yil University, Faculty of Medicine, Department of Otorhinolaryngology, Van, Turkey

**Keywords:** Angiographic embolization, Carotid bifurcation, Cervical paragangliomas, Shamblin classification

## Abstract

**Introduction and objective:**

To report a single center experience with carotid body paraganglioma cases that were treated by the same surgeon in a city with high prevalence of paragangliomas due to high altitude.

**Methods:**

We retrospectively investigated the demographic, clinicopathological and radiological data of 104 patients diagnosed with cervical paragangliomas between 2003 and 2017. The patients were classified according to the Shamblin classification.

**Results:**

In this study a total of 104 patients (33 male and 71 female, with a mean age of 54.6 ± 13 years) diagnosed with cervical paragangliomas located on carotid bifurcation between 2003 and 2017 were included. Among those patients, 10 presented with bilateral tumors and in total, 114 paragangliomas were managed in this period. The mean diameter of the tumors was 5.12 ± 1.45 cm. Malignant tumor was determined in only one (0.9%) patient. All patients were operated. In 12 patients with the tumor diameter larger than 5 cm, preoperative coil embolization was achieved. In 14 patients, preoperative angiographic embolization was employed and in 4 patients intraoperative sclerosing agent injections were performed. Facial paralysis was observed in 2 patients and dysphagia was present in 1 patient, Horner syndrome was seen in 1 patient and hoarseness was reported in 7 patients after operation. All those complications improved during follow-up. Mortality was not reported in any cases.

**Conclusion:**

Surgery is the definitive treatment for patients with cervical paragangliomas. Although, it may be difficult in patients with the advanced Shamblin types, in experienced hands, complication rates are very low.

## Introduction

Paragangliomas are rare, highly vascular neuro-endocrine tumors originating from neural crest cells that can be located anywhere from skull base to the sacrum.^1^ About one-third of paragangliomas are hereditary; a few of them accompanying familial tumors such as multiple endocrine neoplasia Type 2 (MEN 2), von Hippel-Lindau (vHL) disease or neurofibromatosis type.^2^ Metastasis, defined as the spread of tumor to the sites where chromaffin tissue is normally absent, such as lymph nodes, liver, bone, and lungs has been reported in less than 5% of carotid body paragangliomas.^3^ Due to the slow progression of the disease, malignancy is not always associated with a poor short-term prognosis.

About 2/3 of paragangliomas are located in the adrenal gland and remaining extra-adrenal tumors were reported in the abdomen, thorax, and rarely in the head and neck region.^4^ Head and neck paragangliomas generally grow slowly and may remain silent for years.^5^ The most common head and neck paragangliomas are carotid body tumors.^6^ About 70–80% of head and neck paragangliomas are asymptomatic and depending on the location, they may manifest different findings and symptoms, such as painless cervical mass, cranial nerve paralysis, dysphagia and hoarseness, pulsatile tinnitus and hearing loss, or difficulties in speech, swallowing, and airway function.^7^ Carotid tumors grow slowly. Although they are benign in general, they cause symptoms due to compression on neighboring vascular or neural structures. For that reason, their surgical exploration is required. If surgically complete exploration is not possible due to the factors associated with the patient or tumor localization, radiotherapy should be considered. Although carotid tumors are radiosensitive, total resolution with radiotherapy is rare. In general, with radiotherapy, tumor stabilization or partial regression is the goal.^8^

In this study, we will report a single center experience of 114 cervical paraganglioma cases in 14 years that were treated by the same surgeon in a city with high prevalence of paragangliomas due to high altitude.

## Methods

We retrospectively investigated the demographic, clinic-pathological and radiological data of 104 patients diagnosed with and operated for cervical paragangliomas between 2003 and 2017 in Van Training and Research Hospital and Van Lokman Hekim Private Hospital. Postoperative results and operative complications were also recorded.

**Figure 1:**
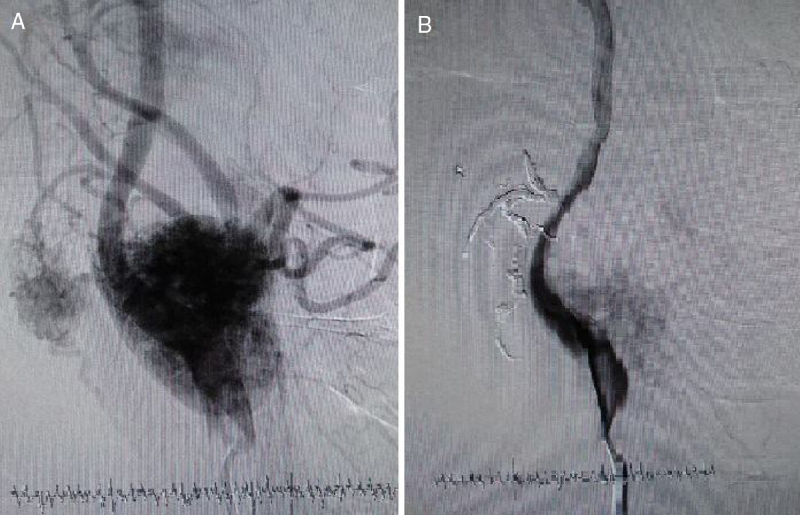
(A) Angiography before coil embolization. (B) Angiography after coil embolization.

**Figure 2:**
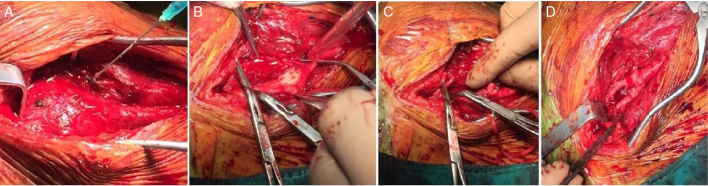
(A) Intraoperative coil embolization. (B) Shamblin Type III patient – Carotid invasion. (C) Division of external carotid artery. (D) Re-anastomosis of division of external carotid artery.

**Figure 3:**
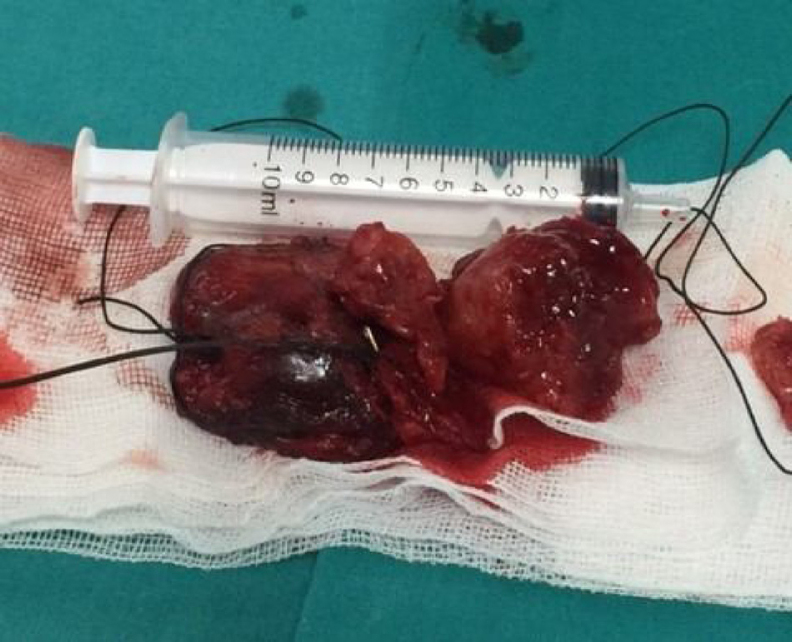
Macroscopic appearance of the tumor.

The patients were classified according to the Shamblin classification.^9^ Only patients operated for cervical paragangliomas were included in the study. In preoperative imaging of the cases, colored Doppler ultrasound and magnetic resonance imaging were performed. In patients who had embolization, carotid angiography was also performed. In all patients with a tumor larger than 5 cm and with high vascularity, preoperative angiographic embolization was performed (Fig. 1). The patients were operated the day after angiographic embolization if it was required. In patients with large amounts of bleeding during operations, intraoperative coils were inserted. In all patients with a tumor larger than 5 cm, division of internal carotid artery was performed and end-to-end anastomosis was achieved after removal (Figure 2, Figure 3). In patients with Shamblin III tumors, if required, the artery was excised and a PTFE graft or saphenous graft inter-positioned. If a graft was required or anastomosis was performed, anti-coagulant treatment was started for 6 months. In patients with a positive family history, annual controls were performed with Doppler ultrasound and magnetic resonance imaging.

### Statistical analysis

The data were analyzed using SPSS 21. Descriptive statistics were performed. Numerical variables were expressed as mean ± standard deviation, and categorical variables were analyzed as frequency and percentage. *p* < 0.05 was considered statistically significant.

## Results

In this study, a total of 104 patients diagnosed with cervical paragangliomas located on the carotid bifurcation, treated between 2003 and 2017, were included. Among those patients, 10 presented with bilateral tumors and a total of 114 paragangliomas were managed in this period. The mean follow-up period was 54 ± 30 months (range: 1–96 months). Among those patients, 33 were male and 71 were female with a mean age of 54.6 ± 13 years (range: 18–83 years). The mean age of males was 56.4 ± 12 years and the mean age of females was 53.1 ± 13 years; there was not any statistically significant difference between genders regarding the mean age at the diagnosis (*p* > 0.05). In 10 (3 male, 7 female) of the patients, paragangliomas were bilateral. In both genders, in about 9–10% of the patients, the tumors were bilateral. Family history was present in 2 (2/10) of the patients with bilateral tumors.

The dimensions of the tumors ranged between 1.3 cm and 10.6 cm with a mean diameter of 5.12 ± 1.45 cm. Malignant tumor was determined in only one female (0.9%) patient at the age of 65 years. In 2 (1.8%) patients, recurrence was reported.

In 15 (19.9%) patients, there was a family history for paragangliomas. In all patients, the main finding was a mass in the cervical region. The patients were grouped according to the Shamblin classification and regarding this classification; 15 (13.2%) were Shamblin Type I, 66 (57.9%) were Shamblin Type II, and 33 (28.9%) were Shamblin Type III.

Surgical methods applied to the patients are summarized in Table 1. In 12 patients with the tumor diameter larger than 5 cm, preoperative coil embolization was achieved. In 14 patients, preoperative angiographic embolization and in 4 patients intraoperative sclerosing agent injections were performed. In 30 patients, division of the external carotid artery was performed and it was re-anastomosed after the removal of the mass. In 4 patients with Shamblin III tumors, internal and external carotid arteries were excised and repaired with PTFE and saphenous vein graft.

**Table 1:** Surgical methods applied to the patients.

Surgical method	Number of patients (%)
Surgical resection	66
Surgical resection and external carotid artery division	30
Surgical resection and PTFE graft interposition	2
Surgical resection and saphena graft interposition	2
Coil embolization and surgical resection	14

The complications after operations were also recorded. Facial paralysis was apparent in 2 (1.9%) patients, dysphagia occurred in 1 (0.9%) patient, Horner's syndrome was seen in 1 (0.9%) patient and hoarseness was reported in 7 (6.7%) patients: those were not persistent in any of the cases. All those complications improved during follow-up. Mortality was not reported in any cases.

## Discussion

In this study, we reported the general characteristics of 104 patients with cervical paragangliomas located on the carotid bifurcation. To the best of our knowledge, this is one of the largest series in the literature reporting the outcomes of cervical paragangliomas.

Female to male ratio was 2.15 in this study. A female predominance was also reported previously in some studies.^10^ However, in a retrospective study on 10 patients, Darouassi et al.^11^ reported a male predominance with a sex-ratio of 2.33. A slow growing, painless mass was the most common clinical presentation in our study as reported before.^11^, ^12^, ^13^ Luna-Ortiz et al.^14^ also reported their 20 years experience on 69 carotid body tumors and determined that 96.9% of the patients were female and the most common presentation was also a painless neck mass determined in 78.7% of patients. In that study, 86.8% of patients were treated with surgery. Pain, dysphonia, dizziness, headache and tinnitus may also be the main complaints at diagnosis.

In preoperative diagnosis, imaging is crucial since the differential diagnosis includes thyroid nodules, lymphadenopathy and brachial cysts. Fine-needle aspiration biopsy is not employed since it has a high complication risk due to the hyper-vascularization of the tumor and moreover the cytological evaluation cannot differentiate benign from malignant lesions. In this study, all patients were diagnosed with the imaging techniques and fine-needle aspiration biopsy was not performed in any patients.

The incidence of familial carotid bifurcation tumors was reported as 20% in previous studies.^15^, ^16^ In this study, the ratio of familial cases was 19.9%, which was compatible with the literature.

Mediouni et al.^17^ analyzed the general characteristics of 131 benign paragangliomas and compared them to 11 malignant paragangliomas cases. They reported that; the benign paragangliomas were mostly observed in women with a mean age of 45 years at time of diagnosis. In that study tympanojugular sites were the most common sites followed by carotid and vagal sites. On the other hand, the malignant tumors were mainly observed in younger patients and they were predominantly carotid tumors. In our study, there was only one malignant paraganglioma in a female patient diagnosed at the age of 65 years. In this case, the tumor was unilateral.

With the development of safe embolization protocols, surgical resection has become the preferred treatment option in cervical paragangliomas.^15^ However, due to its localization near large vascular structures and cranial nerves, the surgical treatment is challenging. The surgery should be as conservative as possible to minimize the complications. In that aspect, preoperative embolization was mainly advised in large and hyper-vascularized tumors.^16^ Jianu et al.^18^ reported the treatment outcomes of 7 patients (5 women, 2 men with a mean age of 54.7 years) diagnosed with cervical paragangliomas, who were all operated without any preoperative embolization. They did not observe any perioperative complications in 6 patients but in 1 case, a transient ipsilateral vagus nerve deficit was reported. There was no sign of recurrences in 3 years of follow-up in that study. Chan et al.^19^ analyzed the treatment outcomes of patients with head and neck paragangliomas in a nationwide survey and reported that 91% of cases were treated with surgery alone, and embolization alone was performed in 4% of cases. Postoperative complications were more common in patients undergoing both embolization and surgery together; while acute medical complications, including acute renal failure and pneumonia, were more likely reported in patients undergoing embolization only. In our study, although we did not compare the patients who were treated with or without endovascular interventions; we did not observe an association. It should also be kept in mind that, in general embolization is required in patients with large tumors and it is not surprising that larger tumors were associated with higher complication rates.

In a retrospective study, Lamblin et al.^20^ evaluated the treatment outcomes in 54 carotid body tumor resections in 49 patients and reported that early (in 1 month after surgery) complications occurred in 31 cases, including 30 cases of cranial nerve deficit (56%). They also reported that; 8 patients (17%) showed no cranial nerve deficit recovery, even after 18 months of follow-up. Dorobisz et al.^21^ analyzed the medical data of 47 patients who were diagnosed with and operated for carotid paragangliomas and reported that in 43 cases (88%), simple resection of the tumor was performed, including 11 cases (22%) that additionally required vascular suturing, and 5 (10%) that required reconstruction of the internal carotid artery. Regarding the postoperative complications, 3 patients (6%) were re-operated because of symptoms of cerebral stroke, hypoglossal nerve palsy was determined in 3 cases (6%), and facial nerve palsy in 2 patients (4%), while postoperative hematomas in the wound was observed in 6 patients (12%). We observed facial paralysis in 2 (1.9%) patients, dysphagia in 1 (0.9%) patient, Horner's syndrome in 1 (0.9%) patient and hoarseness in 7 (6.7%) patients; all those complications were reversible in follow-up.

In this study, in 10 patients, the tumors were bilateral. In bilateral paragangliomas, some risk factors such as genetic predisposition, prior neck surgery or radiotherapy were defined.^22^ Family history was present in 2 of 10 patients with bilateral cervical paragangliomas. Fortunately, with the development of more accurate diagnostic methods, paragangliomas are diagnosed at earlier stages. In our study, 28.9% of paragangliomas were Shamblin Type III and there were not any Shamblin Type IV cases. With an advanced stage, complication risks including nerve injuries also increase. In our study in 7 patients reversible hoarseness was determined that was due to the vagal or hypoglossal nerve injury.

Recently, about 20–30% of head and neck paragangliomas were determined to be genetic and associated with germline mutations.^23^ In especially multicenteric or recurrent cases genetic mutations should be suspected. However, because of the expense, we do not routinely perform genetic tests in daily practice.

## Conclusion

In conclusion, surgery is the definitive treatment in patients with cervical paragangliomas. Although, it may be difficult in patients with the advanced Shamblin types, in experienced hands, complication rates are very low.

## Conflicts of interest

The authors declare no conflicts of interest.
